# Learning the language of science: A pilot study exploring citizen scientists’ identity and communication with researchers

**DOI:** 10.1017/cts.2021.847

**Published:** 2021-09-13

**Authors:** Rachel Damiani, Janice L. Krieger, Debbie Treise, Kim Walsh-Childers, Carla L. Fisher, Shirley Bloodworth, Janet Brishke, Elizabeth Shenkman

**Affiliations:** 1 College of Journalism & Communications, University of Florida, Gainesville, FL, USA; 2 STEM Translational Communication Center, University of Florida, Gainesville, FL, USA; 3 College of Medicine, University of Florida, Gainesville, FL, USA

**Keywords:** Citizen science, interpersonal communication, qualitative methods, health communication, identity

## Abstract

**Introduction:**

Although the involvement of citizen scientists in research can contribute to scientific benefits, much remains unknown about participants’ lived experiences in research. Thus, the purpose of this study was to explore how citizen scientists describe their role in, motivation for, and communication with researchers.

**Methods:**

In-depth interviews (*N* = 9) were conducted with citizen scientists at a translational health research center.

**Results:**

Key results include that citizen scientists were invested in learning researchers’ discipline-specific language and viewed small group sizes as conducive to their active participation.

**Conclusions:**

Programs can apply these findings in an effort to improve citizen scientists’ long-term engagement in research.

## Introduction

Since 2006, the Clinical and Translational Science Award (CTSA) Program has recognized the importance of community engagement, such as citizen science programs [1,2]. Citizen science research occurs when members of the public engage in the scientific process, including assisting researchers with data collection or providing lay expertise on the study’s design [3,4]. While the term “citizen science” is pervasive in ecology-related research, citizen science efforts have increasingly expanded into a myriad of disciplines, including health research [5–7]. Involving citizen scientists in research has contributed to scientific benefits, including the collection of data and publication of scientific findings [8,9].

Given the importance of these outcomes, scholars are devoting attention to understanding how to mobilize citizen scientists and sustain their long-term involvement [10,11]. For example, past research has examined citizen scientists’ motivations for engaging in research and attitudes toward science [12,13]. However, previous studies have not yet extensively focused on how the identities of citizen scientists may shape communication with researchers as well as the research experience. Thus, the purpose of this study was to explore how citizen scientists describe their perceived role in, motivation for, and communication with scientists in translational health research.

## Literature Review

### Citizen Scientists’ Participation in Translational Health Research

Citizen scientists often participate in ecological and conservation studies; however, their involvement is not limited to these disciplines [6,7,14,15]. Researchers have emphasized the potential value of involving citizen scientists in health research [6,7]. Within health research, members of the lay public who assist with research are often referred to by a variety of terms, including community-based research participants (CBRP) or community health workers (CHW) [16–18]. Although the overarching role of contributing to the research process is shared across CBRP, CHW, and citizen scientists, there is arguably a nuanced difference in the operationalization of these terms that is worth noting [4,16–18]. Specifically, a stakeholder’s focus on a specific disease or community concern is often emphasized as salient factors in CBPR and CHW partnerships [16–18]. In contrast, citizen science in the translational health context can be viewed from a more holistic lens, in which individuals’ broad “life experiences” (e.g., former profession and role in their family) may provide helpful insight to scientists as they conduct their research [19, p1].

The current pilot study focuses on an active citizen science program at a large CTSA in the southeast, which was created to forge collaborations between public participants and scientists to improve the translational health research process. While CTSAs have different names for programs and initiatives that engage community members [15], this program is self-named by the participants who viewed the title “citizen scientist” as the most appropriate reflection of their role. By bringing their own life experiences and personal expertise to the research setting, citizen scientists may improve the quality and relevance of research and assist scientists with translating findings into a variety of health care settings. Throughout their time in the program, citizen scientists met regularly with a research coordinator who provided training in foundational aspects of the research process (e.g., informed consent and reading a research study). These meetings provided space for citizen scientists to network with one another and gain insight about research basics that could assist them during their meetings with researchers.

In addition to these regular training meetings with the research coordinator, citizen scientists were assigned to various research projects, depending on the needs of different departments and investigators. The number of citizen scientists working on each research project – and the duration of their involvement – varied depending on the study. For instance, two citizen scientists may meet with a research team separately from their training meetings to provide feedback on dissemination materials. Another citizen scientist may be assigned to a specific center within the university and provide a critical lay perspective at their regularly occurring meetings. Citizen scientists could work on one or more research studies with varying levels of engagement. Specifically, a citizen scientist’s involvement in research could span from minimal engagement (e.g., providing one-time feedback on a grant application), to moderate (e.g., assisting with the development of materials to be used to recruit the target population), to sustained (e.g., being a co-author on a research study) [19].

### Study Purpose and Research Questions

When performing their roles, citizen scientists who may have limited scientific backgrounds interact with scientific experts who speak a nuanced, discipline-specific language. In part due to these differences, Aikenhead describes interactions between researchers and non-scientists as a “cross-cultural event” [20, p23]. As such, interactions between citizen scientists and researchers can be considered intergroup interactions, which require stakeholders to negotiate differences in their language and identity [21]. Despite the importance of community engagement for advancing translational health goals, much remains unknown about citizen scientists’ experiences interacting with researchers in this context. To this end, we sought to better understand citizen scientists’ perceptions of their motivations in research and communication with scientists. Below the specific research questions are delineated.

**RQ1** – What motivates citizen scientists to participate in research?

**RQ2** – How do citizen scientists perceive their role in research?

**RQ3** – What factors influence citizen scientists’ interactions with researchers?

## Methods

### Sample and Procedures

Upon approval from a (blinded) institutional review board, the first author contacted all members of the citizen science program via email to participate in semi-structured, in-depth interviews. A total of 12 current or former citizen scientists were invited to participate in semi-structured, in-depth interviews. Of those recruited, nine citizen scientists chose to participate in the study. Eight of these participants were active members of the Citizen Scientist Program, while one participant was a former member of the program. Participants (*N* = 9) ranged in age from 20 to 87 years old (*M* = 53), and five participants were 65 years of age or older. Of the nine participants, five were Caucasian, two were Hispanic or Latino, and two were Black or African American. Participants received a $20 gift card to compensate them for their time.

The first author interviewed the nine recruited citizen scientists from the program and conducted follow-up interviews with six of these citizen scientists. All interviews were audio-recorded and conducted in-person in a public setting to ease the burden for participants. Initial interviews ranged from 42 to 93 minutes (*M* = 63), and follow-up interviews had a slightly shorter range from 26 to 55 minutes (*M* = 43). After participants consented to the interview, they were asked to describe their role in the program, their motivation to participate, and their experience communicating with researchers, following a semi-structured interview guide. The first author transcribed all interviews resulting in a data set of 552 double-spaced pages of transcripts. To maintain confidentiality, we assigned each participant a pseudonym during transcription.

### Data Collection and Analysis

The interview guide was developed to reflect theoretical tenets of the Communication Theory of Identity (CTI). The CTI posits that a person’s identity is created through communication, or the exchange of verbal and non-verbal messages, and exists across four frames: 1) personal (how one perceives his/her own sense of self); 2) enacted (how one performs this sense of self through interactions with others); 3) relational (how one expresses identity through relationships with others; and 4) communal (how one’s identity stems from membership in a community) [22]. With the CTI in mind, we sought to explore the personal and relational frames of identity experienced by citizen scientists, with RQ1 and RQ2 relating to aspects of the personal frame and RQ3 narrowing in on the relational frame.

Data analysis was conducted according to thematic analysis procedures outlined in Braun and Clarke [23]. The first author wrote and reviewed memos across the research process and familiarized herself with the transcripts. After each interview was transcribed, the first author assigned broad conceptual categories through line by line analysis [23]. In line with similar studies, the unit of analysis for coding was a complete thought [24]. Open codes were created in vivo when possible to keep participants’ voices at the forefront of interpretation. Throughout data analysis, the first author used the personal and relational frames of identity from the CTI as sensitizing concepts to better understand participants’ experiences [22,25]. Using constant comparison techniques, open codes were collapsed or eliminated to develop core themes and subthemes [26].

Rigor was maintained in this study through a multi-faceted set of procedures, including maintaining reflexivity and methodological coherence [27], conducting follow-up interviews to hone theoretical sensitivity [28], member checking [29], negative case consideration [27], and working with a second coder to verify the codebook. Throughout the data collection and analysis process – which occurred concurrently – the first author wrote memos to ensure the research questions, data collection, and data analysis were aligned (i.e., methodological coherence) [27]. Additionally, she conducted follow-up interviews with six citizen scientists to refine her understanding of emerging codes and develop a holistic understanding of participants’ experiences [28]. Another verification strategy used throughout data analysis was actively looking for instances in which participants expressed ideas or experiences contrary to emerging themes (i.e., negative case analysis) [27]. For example, one participant expressed an extrinsic factor (i.e., financial compensation) as a main motive for research engagement, which broadened understanding of the range of reasons for why citizen scientists may participate in research depending on their specific needs and interests.

In addition, member checking was employed to ensure participants’ voices and experiences were not misinterpreted [29]. Specifically, the preliminary findings were shared with a subsample (*n* = 7) of citizen scientists at one of their regularly occurring meetings, followed by refinement of themes. During data analysis, the first author worked with a second coder to verify the qualitative data analysis. After developing a codebook and analyzing the data, the first author trained a second coder by reviewing two transcripts line by line with the initial codebook. Throughout this training process, the two coders discussed any differences in their interpretations of the codebook or data. Differences were resolved via in-depth discussions and adjustments of the codebook.

After this training period, both coders then independently coded six additional transcripts using this updated codebook. The two coders then reviewed each of these six transcripts, one at a time, together. They discussed any differences in their coding, resolved differences via discussion, and refined the codebook to capture the resolutions. For example, the coders exhibited differences in their coding of two properties within the “Researcher’s Inclusivity & Connectivity” theme, which led to discussions about the analytical overlap between these two properties and a resolution collapsing these properties into one subtheme, “inclusive personality.” This codebook verification process was completed twice: first to verify the major themes (e.g., “engaging with researcher to assist in research translation”) and second to verify subthemes (e.g., “directly connecting scientists with the community”). After completing this verification process and refining the codebook, the first author recoded all of the transcripts in NVivo using the updated codebook.

## Results

### RQ 1: What Motivates Citizen Scientists to Participate in Research?

#### Personal Enjoyment/Interest

Citizen scientists described being motivated to participate in research because of their personal enjoyment of or interest in research (see Table 1 for frequencies of codes for RQ1). Specifically, they relayed that citizen scientist participation connected with aspects of their personal identity related to research participation, including a love of learning and enjoyment of science. For example, when asked about his motivation to be a citizen scientist, Fred said, “I find the work interesting.” Another citizen scientist, Taylor, said her interest in being a citizen scientist was piqued because of “the thought of being able to learn something new.” While the majority of citizen scientists described an intrinsic motivation to participate in research because they found the activity interesting or enjoyable, one participant, Alicia, relayed that she initially had decided to become a citizen scientist for extrinsic reasons (i.e., financial compensation).

**Table 1.** Frequencies of codes for RQ1: What motivates citizen scientists to participate in research?

Code	Frequency of code									
	Alicia	Courtney	Fred	Jane	Jess	John	Kate	Mary	Taylor	Overall
Personal enjoyment/interest	2	11	4	2	2	2	6	6	10	45
Beneficial activity	1	4	0	7	2	6	3	2	2	27
For the individual	1	2	0	5	2	2	3	0	1	16
For the community	0	2	0	2	0	4	0	2	1	11

#### Research as a Beneficial Activity

Many citizen scientists expressed that they were motivated to participate in research because they viewed it as a worthwhile activity that could benefit their *individual life* or their *community*. For example, John described how one of his motivations for participating in research was improving his own health. He said, “Getting closer to the research community may lead to contacts that I can follow up on with regard to my personal health issues.” Similarly, Jane expressed that her motivation to participate stemmed from wanting to change her own health and the lives of family members close to her. Additionally, citizen scientists relayed that they were driven to be a part of a worthwhile activity that could *benefit their community* or the scientific community at large. John said, “I thought this would be a great opportunity to get involved and hopefully see some changes in the way research is conducted, presented, implemented.”

### RQ 2: How Do Citizen Scientists Perceive Their Role in Research?

#### “Bridging the Gap” Between Scientists and the “regular Joe”

Multiple citizen scientists described how they viewed their role as a citizen scientist as bridging the scientific and lay communities (See Table 2 for frequencies of codes for RQ2). Specifically, some citizen scientists *conceptualized a bridge identity* by explaining how they functioned as the bridge between community members and scientists. Citizen scientists also described two main ways in which they “bridged the gap” by *engaging with 1) researchers and/or 2) community members to assist in research translation*.

**Table 2.** Frequencies of codes for RQ2: How do citizen scientists perceive their role in research?

Code	Frequency of code									
	Alicia	Courtney	Fred	Jane	Jess	John	Kate	Mary	Taylor	Overall
"Bridging the Gap" between scientists and the "regular Joe"	8	16	12	10	9	13	10	16	9	103
Conceptualizing a bridge identity	1	0	0	1	1	0	0	4	0	7
Engaging with researchers	7	11	11	5	7	10	10	8	6	75
Relaying the interests and concerns of the "regular Joe" to scientists	7	8	11	5	7	9	10	7	6	70
Directly connecting scientists with the community	0	3	0	0	0	1	0	1	0	5
Engaging with community members	0	5	1	4	1	3	0	4	3	21
Explaining or defining citizen science	0	3	0	0	0	0	0	1	1	5
Sharing information with the community	0	2	0	4	0	0	0	1	1	8
Gathering community members’ stories and concerns	0	0	0	0	0	1	0	1	1	3
Aspire to engage with the community	0	0	1	0	1	2	0	1	0	5

#### Conceptualizing a Bridge Identity

One citizen scientist, Jane, summarized this *conceptualizing a bridge* idea when she said, “I think my role has been a bridge, as far as bridging the gap between the professional research and the everyday ordinary person.” Similarly, Alicia expressed, “We are the people who help scientists make their research more accessible to everyday people.” Rather than using the term “bridge,” Jess described a citizen scientist’s role as being a “liaison” to describe how citizen scientists can operate at the interface of science and society. The next two subthemes provide more specific ways in which citizen scientists try to enact this bridge role by focusing on engaging with researchers and community members to assist in research translation.

#### Engaging with Researchers to Assist in Research Translation

Citizen scientists described engaging with researchers to assist in research translation in two main ways: *directly connecting scientists with the community* and *relaying the interests and concerns of the “Regular Joe”* to scientists. Specifically, some citizen scientists said they may help connect researchers with the community. For example, Mary relayed how she can assist investigators with recruitment for research studies: “I think that the research that involves people becoming subjects, we have a lot of value and the value is making that bridge and also talking to the researcher about how they might approach people.” Besides recruitment, citizen scientists described how they worked to relay the interests of the “Regular Joe” by providing feedback on research materials and representing the average person. For example, Jess expressed that a citizen scientist’s role is to provide researchers with “good feedback when things might seem to the layperson a little bit confusing.”

While multiple citizen scientists described providing feedback on research proposals, other citizen scientists shared insight with researchers about different research materials, including a phone app that was being developed to assist patients with a health condition. In addition to voicing the concerns of the community, citizen scientists also described a role in representation. For example, Jess described how they viewed their role as representing the lay public to scientists by being “at the table when research is happening.” Another way in which some citizen scientists described viewing their role in representation was by providing the “human element” in research. For example, Courtney said, “research is black and white, and we kind of throw some color into it.”

#### Engaging with Community Members to Assist in Research Translation

Citizen scientists’ perceived roles and enacted identity were not limited to interacting with researchers; many participants described engaging with community members to assist with research translation in three main ways: *explaining or defining citizen science, sharing information with the community*, and *gathering community members’ stories and concerns*. Some participants described *explaining or defining citizen scientist* to community members who were unfamiliar with the term. For example, Courtney said, “I’ve been involved with helping people understand that there are citizen scientists out there and how they can relate with us.” In addition to explaining the concept of citizen science, participants relayed that they shared *scientific information with community members*. For example, Jane described how she communicated information about nutrition to members of her community.

Another way in which some citizen scientists described enacting their identity as a citizen scientist was *gathering community members’ stories and concerns*. Mary said, “I see my job as listening to and trying to identify those problems and helping in any way that I can.” Similarly, John relayed how he listens to the stories of those around him about “health-related issues.” He said, “There’s dozens of stories out there like this, and I think the more we can put the human element on these statistics, the more understanding we will get of at what’s at stake here.” In addition to these three different ways of engaging with community members, some citizen scientists described *aspiring to engage with community members*. These citizen scientists said that they hoped to interact more with the community in future tasks. For example, Jess said, “We were talking about how can we get the citizen scientists more in our community and be utilized?”

### RQ3: What Factors Influence Citizen Scientists’ Interactions with Researchers?

#### Group Size

Many participants described how the size of the group influenced their ability to enact their identity as a citizen scientist (See Table 3 for frequencies of codes for RQ3). Specifically, some citizen scientists relayed that group size influenced their interactions with researchers for three main reasons: *opportunities for engagement*, *ability to focus and understand the content*, and *intergroup dynamics*. Specifically, some citizen scientists described how smaller group sizes provided more *opportunities for engagement*. For example, Fred described attempting to engage with researchers in a large group setting. He said: “You’ve got two conference rooms, two screens, and dozens of researchers all vying to ask questions, so your participation is limited just by the sheer numbers of people involved.” Additionally, some participants described how a smaller group size was more conducive for facilitating their *ability to focus and understand the content*. For example, Kate described how she would sometimes “check out” in large groups. In contrast, she expressed that when she meets with researchers in small groups or one-on-one, she has more time to understand the material and “talk it through” with the researcher.

**Table 3.** Frequencies of codes for RQ3: What factors influence citizen scientists’ interactions with researchers?

Code	Frequency of code									
	Alicia	Courtney	Fred	Jane	Jess	John	Kate	Mary	Taylor	Overall
Group size	3	5	2	2	1	5	5	1	2	26
Opportunities for engagement	0	3	1	1	1	4	1	1	0	12
Ability to focus and understand the content	1	0	1	0	0	0	3	0	0	5
Intergroup dynamics	2	2	0	1	0	1	1	0	2	9
Preparedness	1	5	3	0	5	4	4	3	1	26
Clarity of roles	1	4	2	0	3	1	1	2	1	15
Materials/information beforehand	0	1	1	0	2	3	3	1	0	11
A researcher’s inclusivity & connectivity	3	11	8	13	2	9	7	14	2	69
Inclusive personality & personal connection	2	8	6	12	2	7	7	7	2	53
Sustained interaction	1	3	2	1	0	2	0	7	0	16
Citizen scientists’ investment in learning a researcher’s discipline-specific language	2	5	4	8	4	3	5	7	3	41
Practical steps to learn a researcher’s language	1	1	2	3	1	2	3	2	2	17
Perceived challenges with learning a researcher’s language	0	1	0	1	2	0	0	1	0	5
Open-minded to learning a researcher’s language	0	1	0	2	0	0	2	2	1	8
Motivation to perform perceived role	1	1	2	1	1	1	0	1	0	8
Motivation to reduce intergroup divide	0	1	0	1	0	0	0	1	0	3

In addition to impacting their ability to focus, some citizen scientists commented on how the group setting influenced aspects of the *intergroup dynamic*. Specifically, a few citizen scientists relayed that they were cognizant of the divide between researchers and non-scientists in the large group setting. For example, Alicia said, “researchers are there using big terms and had PowerPoint presentations… so that was a little bit intimidating.” Taylor also noted that the large group setting can influence her willingness to engage with researchers because of her “nerves sometimes.” She elaborated, “Sometimes I’ll think things, but then I’ll feel like maybe it’s wrong, so I don't really say too much, but I’m kind of trying to get away from that.”

#### Preparedness

Some citizen scientists said that feeling prepared for the meeting in two main ways – *clarity of roles and materials/information beforehand* – helped to increase their comfort levels for contributing in the group setting. Specifically, some citizen scientists described being unsure of their specific roles (e.g., *clarity of roles*) when they first met with researchers. Alicia summarized this idea: “I didn't always know what they expected from me.” Fred elaborated on this idea of *clarity of roles*, explaining that he sometimes felt uncertain about how he was supposed to be providing feedback to researchers. He said, “Sometimes I think what hinders my ability to contribute is not being sure that I’ve asked all the questions that I should have been asking.”

In addition to clarity of roles, some participants described how having *materials/information beforehand* facilitated their interactions with researchers. Courtney said that having information before a meeting with researchers can help her “be better informed, and I’d be more comfortable with knowing what was going on…so I think it would be kind of a time saver and it would help us focus.” Besides being able to read these materials ahead of time, one citizen scientist described receiving information before meeting with the researcher as evidence of the researcher’s sincere interest in citizen scientists’ input. Mary said, “whether you get the material beforehand to look at means they’re really asking for some feedback…it’s how they anticipate meeting with you as much as how you anticipate meeting with them.”

#### A Researcher’s Inclusivity and Connectivity

Citizen scientists describe the influence of a researcher’s welcoming personality and connected behavior on perceptions related to their relational identity. Specifically, some participants relayed that a researcher’s *inclusive personality* and *sustained interaction* were integral aspects of the relationship. Many citizen scientists described positive experiences interacting with researchers who were inclusive and engaging. For example, Taylor described researchers she has worked with as “open and down to earth.” Jess also conveyed that many of the researchers were “willing to listen.” In addition, Jane relayed her positive experience with a researcher below.

He’s just got such a wonderful personality and inclusive personality and he, not saying others don't, but he has a connection with me in my mind because he can flip from researcher-doctor and get right on the level – I’m not saying come down – but transfer to the level of talking to a person…and making you feel like a viable part of the team.

In addition to a researcher’s inclusivity, some citizen scientists described that they enjoyed when scientists developed a *personal connection* with them. For example, Kate relayed that she “appreciates being able to talk to them about life.” She said, “Once you break behind that barrier of researcher, they’re just people too, and it’s a lot easier to feel more comfortable in the role of a citizen scientist.” Some participants also relayed the influence of a *sustained interaction* (e.g., a scientist providing feedback about the impact of their contributions and long-term interactions with scientists instead of single meetings) on their communication with scientists. For example, Fred summarized that in longer-term interactions with researchers, “barriers do breakdown.”

While many citizen scientists had positive interactions with researchers, some participants described communicating with less inclusive or connected scientists. For example, Mary relayed that she had wanted more feedback about the status of a project from a lead scientist, including the impact of citizen scientists’ feedback, but she also recognized the limitations to this long-term connection. She said, “I would just like to be more in the know about what is happened to the things that I did participate in…and I think if we were in the research community, like passing in the halls, I would know it.” Additionally, John recalled attending a meeting and feeling that the researchers “were really talking among themselves.”

#### Citizen Scientists’ Investment in Learning Discipline-Specific Language

Citizen scientists described being invested in learning a researcher’s discipline-specific language by taking *practical steps to learn a researcher’s language*, working to overcome *perceived challenges with learning a researcher’s language*, and being *open-minded to learning a researcher’s language*. Many participants described *practical steps* that they used to try to better understand a researcher’s discipline-specific language. These strategies ranged from looking up terms on their own to asking questions during meetings. For example, Fred said, “I think in general, if one isn't hesitant about asking for clarification, a lot of it is quite understandable.” Kate also said that new terms “always sounds like gibberish at first, so it doesn't overwhelm me.” When she does not understand the language and is meeting with a researcher one-on-one, she said, “a lot of times you can talk it through.” In addition to asking researcher questions, citizen scientists independently embraced scientist’s language to enact their identity as a citizen scientist. For example, Jane said that she also reads on her own to enable her to be conversant with scientists.

As a citizen scientist, I think that I’m learning to listen more intently, do more research. I’m finding out I have to do a lot more research on my own. I have to do a lot more reading on my own. I’m trying to remember acronyms.

While citizen scientists described being invested in learning a scientist’s language, they also relayed that this task could be challenging. Some citizen scientists described *perceived challenges with learning a researcher’s language* that either the participant him-/herself or a hypothetical citizen scientist might encounter. For example, Jane noted that it can be challenging trying to read scientific articles on her own. She said, “Sometimes I have to go back and read them two or three times to try and understand them.” Even though these challenges can be present, citizen scientists described being *open-minded to learning a researcher’s language*. For example, Mary said, “I’m not intimidated by researchers or by knowledge or by anything. I’m not intimidated by big words, and I like dynamic things.”

#### Motivation to Perform Perceived Role

Citizen scientists described two main motivations guiding their willingness to learn a researcher’s language. First, some citizen scientists described being motivated to learn a researcher’s language to *perform their perceived roles*. For example, Fred relayed that familiarizing himself with a scientist’s language “makes it more possible to be engaged.” Similarly, John said that gaining insight into a researcher’s language “make(s) the role of providing constructive feedback more efficient and effective.” Jane elaborated on how learning a researcher’s language can assist her with providing feedback to researchers during meetings; she said it helps her “interact intellectually or efficiently without just being like an empty wagon, doing a lot of talking but saying nothing.”

#### Motivation to Reduce Intergroup Divide

The second motivation reported by some citizen scientists was their desire to increase their ability to relate to and communicate with researchers (e.g., *reduce their intergroup divide*). For example, Mary said, “Every field has its jargon and if you’re going to relate to people who use jargon, you’ve got to have an understanding of their jargon.” Similarly, Courtney said that if she did not grasp a scientist’s language, she might feel distant from the group.

If I don't know what people are saying, if I don't understand what they’re talking about, then I can't be engaged. I’d feel like alienated, so I need to be able to speak the language to relate with them (Courtney).

## Discussion

The purpose of this qualitative study was to explore how citizen scientists perceive their role in, motivation for, and communication with scientists in research using the CTI as a theoretical lens [22]. This study generated preliminary findings about citizen scientists’ perceptions of their identity in research that are important to highlight. First, these results suggest that citizen scientists invest in learning scientists’ discipline-specific language. This finding is interesting in light of the CTI because it suggests that citizen scientists may strive to create a relational identity in research that is rooted in a shared language with scientists [22]. However, much is unknown about how scientists perceive citizen scientists, including whether they view citizen scientists as willing to embrace a two-way dynamic and learn their language. Thus, future research could investigate scientists’ personal and relational identities to better understand the degree to which members of this dyad are aligned in their perceptions of one another.

Second, our results provide insight about citizen scientists’ personal identity in research, including that many individuals were motivated to participate in science because they viewed the endeavor as enjoyable and beneficial to themselves and/or their communities. These findings are similar to results from past studies indicating that other lay individuals who are involved in research, including potential clinical trial participants [30] and citizen scientists who collect data related to air quality using their phones [31], are motivated to engage in research by the prospect of personal gain and/or contributing to society. It is important to note that individuals in the present study are unique from both of these aforementioned contexts in that they are not clinical trial participants, nor are they participating in research via their smartphone solely [30,31]. Rather, citizen scientists in this study engage with researchers and one another through meetings in which they provide lay expertise and feedback [19].

Thus, it would be beneficial for future research studies to further explore the spectrum of lay individuals’ motivations to engage with researchers, along with nuanced differences across discipline and context. This insight about lay members’ personal identity, including their motivations to participate in research, could assist program coordinators and scientists in designing their programs to ensure that participants have an enriching experience. For example, programs could provide additional learning opportunities for citizen scientists outside of regular research meetings to connect with their personal interest and enjoyment in research. This tailoring of the research experience to align with individuals’ motivations and interests could help sustain citizen scientists’ participation and assist with recruitment.

### Practical and Theoretical Implications

Citizen scientists in this study described multiple factors that influenced their enactment of their research identity, including feeling more comfortable engaging with researchers in small groups and having materials about the research ahead of time. Moreover, citizen scientists described the positive impact of a researcher’s connectivity and inclusivity, including having a sustained interaction with a scientist to build rapport. These preliminary findings connect to practical suggestions that programs can implement in an effort to facilitate citizen scientists’ engagement with researchers. In future studies, researchers could quantitatively examine the influence of group size and preparedness on self-reported engagement and communication satisfaction with researchers. This line of research could contribute to a growing understanding of the communicative dynamics underlying community engagement [32] and lead to practice-based recommendations.

This study’s preliminary findings also sparked theoretical inquiries related to the finding that citizen scientists viewed one aspect of their personal identity in research as bridging the gap between scientists and members of the lay public. This idea of serving as a “bridge” between two social groups connects with the concepts of cultural brokering and intergroup communication accommodation in translating science [21,33]. Interestingly, while participants in this study had a variety of backgrounds (e.g., age, ethnicity, profession), all citizen scientists expressed that they adopted one or more aspects of this bridge identity in research. This finding suggests that, regardless of some of their differences, citizen scientists may forge a communal identity with one another based on their role in research [22]. Thus, programs can create opportunities for citizen scientists to bond with one another outside of their meetings with researchers, such as this program, which invested in regular training meetings. Moreover, future studies could build upon these pilot findings to examine: 1) whether citizen scientists across contexts adopt a cultural broker-based identity and 2) the effects of citizen scientists’ perceived identity and communication accommodation on outcomes (e.g., longevity of citizen scientists’ participation).

### Strengths and Limitations

This study’s limitations include its small number of participants from one citizen science program in a single geographic area. As additional citizen science programs in translational health research arise and this current one expands, we hope to conduct additional mixed-methods studies with a larger sample size across programs. In these future efforts, it would be critical to explore how other aspects of participants’ background and identity (e.g., age, gender, and ethnicity) may influence how citizen scientists perceive their roles and motivations in research. For instance, the citizen science program in this study has, in general, more difficulty recruiting men and younger participants into the program, which aligns with past research suggesting that women and older individuals have greater participation in volunteer efforts [34]. Future quantitative research studies could examine whether the identities documented in the present study (e.g., bridge identity) are related to other potential aspects of an individual’s identity or background, including gender norms (e.g., females as relationally oriented in their communication with others) [35], and attitudes towards science (e.g., mistrust in research among some underrepresented groups) [36].

This pilot study has multiple strengths that are important to note. From a theoretical standpoint, this study brings the CTI [22] into the largely under-explored context of citizen science research. These pilot findings promote understanding of the breadth and utility of this identity lens across contexts, and our findings suggest the context of citizen science is ripe for a communicative identity-based approach. Additionally, although citizen science is expanding into a variety of disciplines, the body of research surrounding citizen science often focuses on the ecological and conservation sciences [14,15]. Thus, another strength of this pilot study is that its findings target the experiences of citizen scientists in an understudied but important field for inquiry: translational health research. Future research efforts can build upon these initial findings to better understand how to leverage citizen scientists’ engagement in translational health research.
